# Progressive Adaptation of a CpGV Isolate to Codling Moth Populations Resistant to CpGV-M

**DOI:** 10.3390/v6125135

**Published:** 2014-12-22

**Authors:** Benoît Graillot, Marie Berling, Christine Blachere-López, Myriam Siegwart, Samantha Besse, Miguel López-Ferber

**Affiliations:** 1 LGEI, Ecole des Mines d’Alès, Institut Mines-Telecom et Université de Montpellier Sud de France, 6, Avenue de Clavières, 30319 Alès, France; E-Mails: benoit.graillot@mines-ales.fr (B.G.); mberling@crea.fr (M.B.); 2 Natural Plant Protection, Arysta LifeScience group, Avenue Léon Blum, 64000 Pau, France; E-Mail: samantha.besse@arysta.com; 3 INRA, 6, Avenue de Clavières, 30319 Alès, France; E-Mail: christine.blachere-lopez@mines-ales.fr; 4 INRA, Unité PSH, Agroparc, 84914 Avignon Cedex 9, France; E-Mail: myriam.siegwart@avignon.inra.fr

**Keywords:** *Cydia pomonella granulovirus*, coddling moth, resistance development, biological control

## Abstract

The NPP-R1 isolate of CpGV is able to replicate on CpGV-M-resistant codling moths. However, its efficacy is not sufficient to provide acceptable levels of control in natural (orchard) conditions. A laboratory colony derived from resistant codling moths was established, which exhibited a homogeneous genetic background and a resistance level more than 7000 fold. By successive cycles of replication of NPP-R1 in this colony, we observed a progressive increase in efficacy. After 16 cycles (isolate 2016-r16), the efficacy of the virus isolate was equivalent to that of CpGV-M on susceptible insects. This isolate was able to control both CpGV-M-susceptible and CpGV-M-resistant insects with similar efficacy. No reduction in the levels of occlusion body production in susceptible larvae was observed for 2016-r16 compared to CpGV-M.

## 1. Introduction

Codling moth (CM) is a major insect pest of apples and pears [1]. CM is distributed worldwide in temperate climates in the majority of apple production areas [2]. After years of chemical control, CM progressively developed resistances to most chemical insecticides [3], making the use of alternative methods for pest control necessary to sustain production and reduce environmental impacts. 

Among biological control agents, baculoviruses (Nucleopolyhedroviruses (NPVs) and Granuloviruses (GVs)) are the only virus group used in field conditions. They are used as microbial insecticides due to their specificity for one or a few insect species [4], in annual cultures, in orchards, or in forests [5,6]. Baculoviruses are double stranded-DNA viruses restricted to invertebrates, and more precisely arthropodes [6]. Baculoviruses have genome sizes ranging from 80–180 kbp, and replicate in the nucleus of infected cells. These viruses are characterised by enveloped rod-shaped nucleocapsids occluded in a proteinaceous matrix [7]. Baculovirus possess two types of particles, the Oclusion Body (OB), containing one or more virions, that ensures horizontal transmission between susceptible hosts; and the Budded Virus, a much simpler particle containing one virus genome used for transmission between cells inside the host. There are two different types of OBs: the first is small, granular, ellipsoidal-shaped occlusion bodies, characteristic of the GVs (now called betabaculoviruses), that generally contains a single enveloped nucleocapsid and the second, bigger, polyhedron shaped, present in all other Baculovirus genera, lepidopteran nucleopolyhedrovirus (NPV) or alphabaculoviruses, hymenopteran NPV or gammabaculoviruses, and dipteran NPV or deltabaculoviruses. Each polyhedron contains numerous nucleocapsids enveloped alone (single enveloped) or in groups (multiple enveloped), and distributed throughout the polyhedral occlusion body matrix. Baculoviruses infect the larval stages of insects particularly in the order Lepidoptera [8].

The first *Cydia pomonella granulovirus* (CpGV) isolate was found in Mexico (CpGV-M, [9]). In Europe, all commercial formulations before 2010 were derived from this CpGV-M isolate [10]. This isolate appears to have limited genetic diversity [11] and a representative clone has been completely sequenced [12], making it the reference genotype.

Since 2004, after years of generalized use, resistance to CpGV-M has been reported in orchards in Germany [13] and France [14]. Now, resistance is distributed across Europe [15], but not in other continents. This may be due to a lower use of CpGV isolates.

In order to bypass this resistance, research was conducted to obtain new viral variants able to control these resistant insect populations. Various natural isolates were found to be able to partially overcome the resistance; among them, the isolate NPP-R1 [16]. 

Natural virus populations present an inherent genetic diversity and are able to evolve when conditions allow adaptation of the pathogen to the host. Taking advantage of this fact, a selection process has been carried out by successive passages on a resistant insect laboratory colony, called RGV, in order to increase the efficiency of the NPP-R1 isolate [16]. The efficiency and specificity of the NPP-R1 virus and the 2016-r4 isolate corresponding to the fourth passage have been previously described. An increasing activity against resistant insects was detected at that point [16]. 

We have continued the process for 16 generations of codling moth. In this study, we characterize the virus populations by their efficacy on controlling CpGV-M susceptible and resistant codling moth laboratory colonies; their genetic composition, and their productivity. 

## 2. Materials and Methods

### 2.1. Insects

Two *Cydia pomonella* laboratory colonies were used in this work, a laboratory colony susceptible to CpGV-M, used as reference colony; and the resistant colony, RGV. This last colony was derived from a natural resistant population (St-A) found in the region of Saint Andiol (Bouches-du-Rhône, France), followed by selection for resistance to CpGV-M as previously described [16]. RGV is periodically selected for CpGV-M resistance in order to maintain a high level of resistance [16].

### 2.2. Viruses

The CpGV-M isolate (our laboratory stock 2020-s1) is used as a reference and comes from the inoculum for production of Carpovirusine (Natural Plant Protection (NPP), Arysta LifeScience group, Pau, France) and is genetically and phenotypically similar to the Mexican isolate discovered and described by Tanada (1964) [9].

The NPP-R1 isolate was provided by NPP. This isolate comes from the virus collection of the firm. The original stock was amplified using the susceptible colony reared at the NPP facility prior to use in bioassays. The 2016-r4; 2016-r8 and 2016-r16 isolates are respectively the result of four, eight and 16 cycles of multiplication of NPP-R1 on RGV larvae.

### 2.3. Amplification of Virus

Third-instar (L3) resistant larvae (7 days old) reared on a virus-free diet were used for viral amplification. A viral suspension was prepared in distilled water at a concentration of 800 OB/µL. Fifty µL was deposited evenly on the surface of a formaldehyde free-diet (Stonefly Heliothis Diet, Ward’s Science, Rochester, NY, USA) in 24-well plates and allowed to penetrate. Then L3 larvae were deposed on these plates and incubated at 25 °C (± 1 °C) with a 16:8-h (light/dark) photoperiod and a relative humidity of 60% (±10%). After 4 days, all larvae presenting signs of viral infection were extracted from the rearing diet and transferred to Eppendorf^®^ tubes and stored at the same temperature for an additional day, then frozen (−20 °C). Larvae were processed as previously described [16]. The occlusion bodies were resuspended in distilled water, and stored at −20 °C.

The same process was carried out for each virus passage.

### 2.4. Evaluation of Viral Production of 2016-r8 and 2016-r16 Isolate

The protocol described above (Section 2.3) was used using susceptible larvae, to allow replication without a selective pressure. Larvae were pooled by groups of three and weighed before homogenisation. 30 groups are analyzed for each isolate. The production yields expressed in OBs/larva and OBs/g of larva were compared using the non-parametric Kruskal-Wallis test.

### 2.5. Bioassays

Bioassays were carried out as previously described [16]. Briefly, neonate larvae of both laboratory colonies, susceptible and resistant (24 h old or less) were individually placed in 96-well plates containing about 200 µL of a formaldehyde-free artificial diet (Stonefly Heliothis Diet, Ward’s Science, Rochester, NY, USA), after a 6-µL volume of an OB suspension was spread over the surface of each well (well surface area = 28 mm^2^). The same volume of distilled water was used in control wells. Bioassays were performed using six virus concentrations, ranging from 2–6250 OBs/µL for the most efficient virus/host combination and 10–100,000 OBs/µL for the least efficient combination. The wells were sealed with Parafilm™, and the microplates were incubated in a growth chamber at 25 °C (± 1 °C) with a 16:8-h (light/dark) photoperiod and a relative humidity of 60% (± 10%). Mortality was recorded at 7 days postinfection. Larvae that did not react to physical stimuli were considered dead. At least three independent experiments were carried out. After verifying their homogeneity, the data were pooled. Mortality data were subjected to probit analysis [17] performed with the POLO+ software (LeOra Software, Berkeley, CA, USA) [18].

### 2.6. DNA Extraction and Restriction Endonuclease (REN) Analysis of CpGV Isolates

Viral DNA of the different productions detailed in Section 2.3, was extracted from OBs as previously described [16]. Approximately 500 ng of purified virus DNA was digested with *Eco*RI, *Bam*HI, *Pst*I, *Sal*I, or *Xho*I (Fisher Scientific, Illkirch, France) in the supplied buffer at 37 °C for 3 h. The DNA restriction fragments were separated by electrophoresis in 1% agarose gels in Tris-acetate-EDTA buffer at 80 V for approximately 4 h. λ-phage DNA/ *Hin*dIII fragments were used as standards for size determination. Fragments were visualized under UV light by ethidium bromide staining. The fragment sizes and DNA quantity were estimated using the Kodak 1D image system (Eastman Kodak Company, Rochester, NY, USA).

## 3. Results

### 3.1. REN Analysis

**Figure 1:**
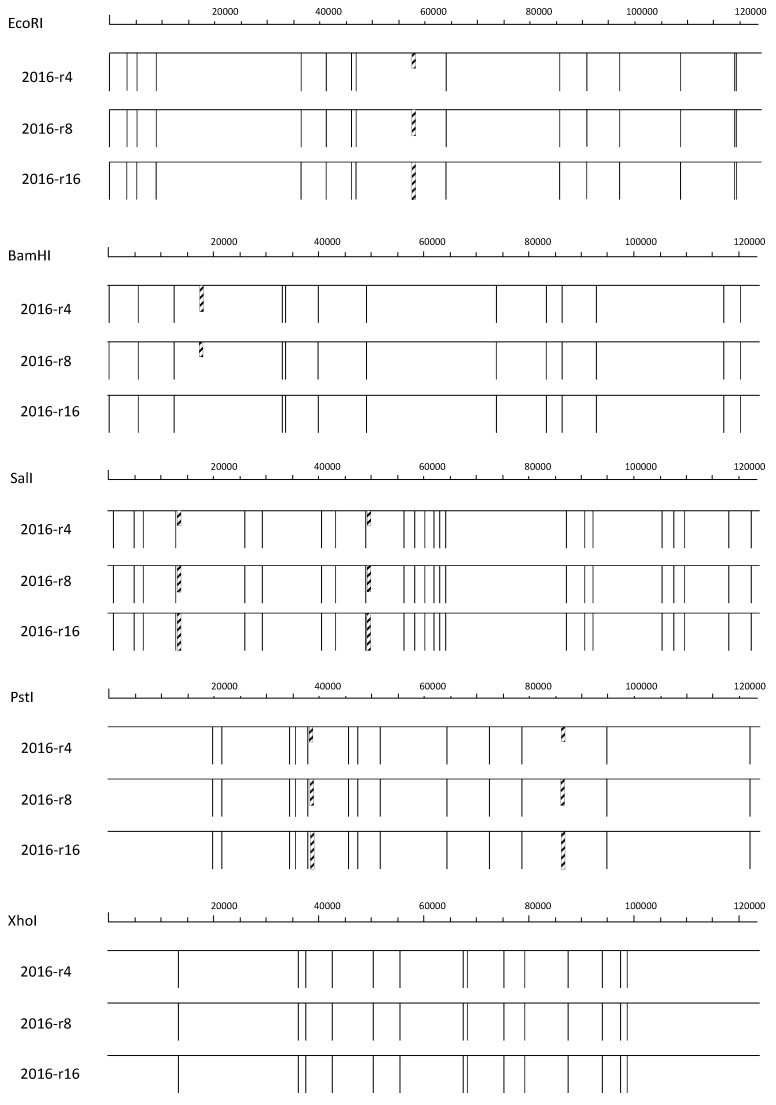
Graphical map of the genomes of CpGV isolates obtained after sequential amplification on resistant RGV larvae. The relative proportion of the variable restriction sites are represented by dashed rectangles. Their length is proportional to the relative abundance. The restriction sites shared by the three isolates are represented by lines of equal length.

DNA from NPP-RI and all the successive passages were compared to that from CpGV-M using *Eco*RI, *Bam*HI, *Pst*I, *Sal*I and *Xho*I. Submolar fragments were observed in all isolates, but their relative proportions changed over passages. The characteristic pattern of CpGV-M, in conformity with the published sequence, was observed for all enzymes in the NPP-R1 isolate, mixed with a new pattern, characterized for a supplementary *Eco*RI site around position 58000, the disappearance of the *Bam*HI site at 17745; the appearance of two *Pst*I sites at 38800 and 86500; for *Sal*I, two supplementary sites were observed around positions 13100 and 49000. No differences were detected on *Xho*I profiles. From passages 4–16, this new profile becomes more frequent. At passage 16, the CpGV-M pattern is undetectable. These changes are represented in Figure 1.

**Table 1:** Production per larvae and per gram of larvae of three virus isolates on susceptible insects.

Virus isolate	Production (per gram of larvae (× 10^11^ OB/g) ± SE	Production per larvae * (× 10^10^ OB/g) ± SE
CpGV-M	4.84 ± 0.29 ^a^	2.48 ± 0.10 ^b^
2016-r8	5.77 ± 0.29 ^a^	2.99 ± 0.19 ^b^
2016-r16	5.41 ± 0.16 ^a^	2.54 ± 0.11 ^b^

* Mean production of a larvae estimated by 10 pools of 3 larvae each. Similar letters indicate no statistically significant differences by Kruskal Wallis test; superscript: results having the same letter. *i.e*., ^a^ or ^b^ indicate they are not statistically different.

**Table 2:** Pathogenicities, measured by LC_50_ and LC_90_, of four virus isolates on *Cydia pomonella* laboratory colonies susceptible and resistant to CpGV-M.

Host colony	Virus isolate	Total No. of insects tested	No. of Occlusion Bodies/µL (95% CI)		Slope ± SE	χ^2^	Resistance factor (fold) ^(a)^	
			LC_50_	LC_90_			LC50	LC90
Susceptible	CpGV-M	786	13.10 (6.55–23.20)	223.10 (110.70–654.18)	1.04 ± 0.09	5.99	1.0	1.0
	NPP-R1 ^(b)^	689	25.80 (14.48–39.93)	328.55 (196.93–702.51)	1.16 ± 0.13	1.28	2.0	1.5
	2016-r4 ^(b)^	999	39.65 (6.40–133.91)	805.85 (260.20–1.36 × 10^3^)	0.98 ± 0.11	13.60	3.0	3.6
	2016-r8	445	48.37 (21.18–81.44)	280.52 (158.02–857.03)	1.68 ± 0.25	4.67	3.7	1.3
	2016-r16	790	6.76 (2.60–13.37)	59.63 (27.54–278.55)	1.36 ± 0.13	11.42	0.5	0.3
Resistant	CpGV-M	396	7.84 × 10^3^ (660.45–4.13 × 10^4^)	1.71 × 10^6^ (2.15 × 10^5^–6.72 × 10^8^)	0.55 ± 0.09	6.32	598	7664.7
	NPP-R1 ^(b)^	578	166.31 (91.21–278.27)	1.28 × 10^4^ (5.95 × 10^3^–3.80 × 10^4^)	0.70 ± 0.08	4.81	12.7	57.4
	2016-r4 ^(b)^	1201	102.31 (63.20–146.91)	1.57 × 10^3^ (1.01 × 10^3^–2.97 × 10^3^)	1.10 ± 0.10	6.21	7.8	7.0
	2016-r8	456	41.27 (26.97–58.96)	319.24 (207.87–582.06)	1.44 ± 0.17	1.83	3.2	1.4
	2016-r16	545	22.43 (13.73–34.36)	410.67 (240.16–846.43)	1.02 ± 0.11	3.60	1.7	1.8

^(a)^ The pathogenicity of CpGV-M on susceptible larvae is used as a reference level; ^(b)^ Results from [16].

### 3.2. Viral Production of Isolates

No difference was found in the production per grams of susceptible larvae between CpGV-M, 2016-r8 and 2016-r16 (Kruskal-Wallis test: χ^2^ = 29, *df* = 28, *p* = 0.4125) (Table 1).

### 3.3. Bioassays

Bioassays were performed with the NPP-R1, 2016-r4 (data from [16]), 2016-r8 and 2016-r16 isolates on susceptible and resistant larvae. The efficiency of each viral isolates was estimated by probit analysis (Table 2).

On susceptible larvae, the NPP-R1, the 2016-r4 and the 2016-r8 isolates were less efficient than CpGV-M isolate. Surprisingly, the 2016-r16 isolate was more efficient (LC_50_: 6.76 OB/µL (2.60–13.37); LC_90_: 59.63 OB/µL (27.54–278.55)) than CpGV-M isolate (LC50: 13.10 OB/µL (6.55–23.20); LC_90_: 223.10 OB/µL (110.70–654.18)). On resistant larvae, an increase of efficiency was observed up to the 2016-r8 isolate. The 2016-r16 isolate had a slightly better LC_50_ (22.43 OB/µL (13.73–34.36)) and similar LC_90_ (410.67 OB/µL (240.16–846.43)) than 2016-r8 (LC_50_: 41.27 OB/µL (26.97–58.96); LC_90_: 319.24 OB/µL (207.87–582.06)) indicating that this isolate was completely stabilized.

## 4. Discussion

Codling moth resistant natural populations did not respond to control by CpGV-M. The resistance levels were variable, from a hundred fold to more than a thousand fold resistance as a function of the relative frequency of the resistant genotypes. The RGV resistant colony, developed from a natural population, exhibits a homogeneous resistance level against CpGV-M of 7665 fold (LC_90_), compared to the level for the susceptible colony. This homogeneity allowed a precise estimate of the resistance, a selection of a virus isolate, and the evaluation of the gains in efficacy over successive passages. The isolate NPP-R1 is partially able to control the resistant colony, but the efficacy ratio (LC_90_ on resistant insect/LC_90_ on susceptible insects = 57) was not satisfactory for its use in field conditions, as it would require application doses too high to be compatible with economic constraints.

Following passages of the NPP-R1 isolate on resistant larvae, the efficiency of the viruses increased up to the 2016-r8 isolate (eighth passage). The efficiency of the 16th passage 2016-r16 was comparable to 2016-r8, indicating a stabilization of the isolate.

A first attempt to characterize the NPP-R1 virus isolate and its evolution over selection was made using a RFLP approach. This approach allowed us to demonstrate that the original isolate is composed of at least two genotypes, one similar to CpGV-M, and that the relative proportion of this genotype diminished over passages on a resistant insect colony, but they do not disappear. The selected genotype is characterized by modifications of various restriction sites on its genome. It has been recently demonstrated that modification at the level of the *pe38* virus gene is responsible of overcoming the resistance [19]. This gene is located between 18574 and 19722 nt on CpGV-M [12]. The differences observed do not affect this region. Accordingly, they probably do not present a selective value.

The final isolate obtained, 2016-r16, was able to efficiently control the resistant colony. Surprisingly, this isolate also controlled the susceptible colony as well or better than the reference isolate, CpGV-M, indicating its potential usefulness as a control agent for insect field populations of codling moth presenting variable ratios of susceptible *vs*. resistant insects.

The OB production under our conditions appeared to be higher than the usual levels (2.5 × 10^9^ OB/larva [20]). The differences were probably related to the protocol of larvae rearing. Using different conditions, Reiser *et al.* (1993) reported a yield of 1.7 × 10^10^ OB/larva [21].

The virus yield was compared on susceptible larvae for 2016-r8 and 2016-r16 and the reference CpGV-M. No differences in virus production were observed either when considering OB per larva nor OB per gram of larvae. The experimental conditions used in our test did not allow verifying if there were differences in the speed of kill.

A recent work revealed that there is no apparent fitness cost for the insect to become resistant [22]. Our data did not find either a cost for the virus to overcome this resistance. Putting together our data, similar efficacy and similar level of production in both insect colonies, it is tempting to hypothesize that there is neither a genetic cost for the virus to overcome the resistance. If there is no genetic cost for a virus population on controlling resistant populations, what is the rationale of the preservation in nature of CpGV-M? Clearly, using only two fitness parameters, it is not possible to accept or refuse this hypothesis, and more research will be required before being able to do so.

The isolate 2016r16 is able to efficiently overcome the resistance to CpGV-M. This isolate is now commercialized by Natural Plant Protection under the “Carpovirusine Evo2” designation.
