# Correction: Graillot, B.; *et al.* Progressive Adaptation of a CpGV Isolate to Codling Moth Populations Resistant to CpGV-M. *Viruses* 2014, *6*, 5135–5144

**DOI:** 10.3390/v7122939

**Published:** 2015-12-03

**Authors:** Benoît Graillot, Marie Berling, Christine Blachere-López, Myriam Siegwart, Samantha Besse, Miguel López-Ferber

**Affiliations:** 1 LGEI, Ecole des Mines d’Alès, Institut Mines-Telecom et Université de Montpellier Sud de France. 6, Avenue de Clavières, 30319 Alès, France; benoit.graillot@mines-ales.fr (B.G.); mberling@crea.fr (M.B.); 2 Natural Plant Protection, Arysta LifeScience Group, Avenue Léon Blum, 64000 Pau, France; samantha.besse@arysta.com; 3 Present address : CREA, 215 Avenue de la Roche Parnale, ZI Motte Longue, 74130 Bonneville, France; 4 INRA, 6, Avenue de Clavières, 30319 Alès, France; christine.blachere-lopez@mines-ales.fr; 5 INRA, unité PSH, Agroparc, 84914 Avignon Cedex 9, France; myriam.siegwart@avignon.inra.fr

**Keywords:** *Cydia pomonella granulovirus*, codling moth, biological control, resistance development

## Abstract

In our article “Progressive Adaptation of a CpGV Isolate to Codling Moth Populations Resistant to CpGV-M.” (*Viruses* 2014, 6, 5135–5144; doi:10.3390/v6125135) [1] we obtained resistance values of the codling moth*, Cydia pomonella,* RGV laboratory colony [2], when challenged with Cydia pomonella Granulovirus, Mexican Isolate (CpGV-M), that were lower than those previously published [2]. Careful analysis of both the RGV colony and the CpGV-M virus stock used led to the realization that a low level contamination of this virus stock with CpGV-R5 occurred. We have made new tests with a verified stock, and the results are now in agreement with those previously published.

In our article “Progressive Adaptation of a CpGV Isolate to Codling Moth Populations Resistant to CpGV-M.” (*Viruses* 2014, *6*, 5135–5144; doi:10.3390/v6125135) [1] we obtained resistance values of the RGV laboratory colony, when challenged with Cydia pomonella Granulovirus, Mexican Isolate (CpGV-M), that were lower than those previously published [2].

Careful analysis of both the RGV colony and the CpGV-M virus stock used led to the realization that a low level contamination of this virus stock with CpGV-R5 occurred.

We have made new tests with a verified stock, and the results are now in agreement with those published by Berling *et al.* [2] and in the same range as those obtained with another insect population, CpRR1 [3].

Below you will find the corrected Table 2 for our recently published article [1], in which line 7 has changed.

**Table 2:** Pathogenicities, measured by lethal concentration (LC)_50_ and LC_90_ of four viral isolates on *Cydia pomonella* laboratory colonies susceptible and resistant to CpGV-M.

Host Colony	Virus Isolate	Total No. of Insects Tested	No. of OB/µL (95% CI)		Slope ± SE	Χ^2^	Resistance Factor (Fold) ^(a)^	
			LC_50_	LC_90_			LC_50_	LC_90_
Susceptible	CpGV-M	786	13.10 (6.55–23.20)	223.10 (110.70–654.18)	1.04 ± 0.09	5.99	1.0	1.0
	NPP-R1 ^(b)^	689	25.80 (14.48–39.93)	328.55 (196.93–702.51)	1.16 ± 0.13	1.28	2.0	1.5
	2016-r4 ^(b)^	999	39.65 (6.40–133.91)	805.85 (260.20–1.36 × 10^3^)	0.98 ± 0.11	13.6	3.0	3.5
	2016-r8	445	48.37 (21.18–81.44)	280.52 (158.02–857.03)	1.678 ± 0.25	4.67	3.7	1.3
	2016-r16	790	6.76 (2.6–13.37)	59.63 (27.54–278.55)	1.36 ± 0.13	11.42	0.5	0.25
Resistant	CpGV-M	1619	2.22 × 10^6^ (1.19 × 10^6^–5.67 × 10^6^)	---	0.50 ± 0.07	10.6	1.7 × 10^5^	---
	NPP-R1 ^(b)^	578	166.31 (91.21–278.27)	1.28 × 10^4^ (5.95 × 10^3^–3.80 × 10^4^)	0.70 ± 0.08	4.81	13	57
	2016-r4 ^(b)^	1201	102.31 (63.20–146.91)	1.57 × 10^3^ (1.01 × 10^3^–2.97 × 10^3^)	1.10 ± 0.10	6.21	7.8	7
	2016-r8	456	41.27 (26.97–58.96)	319.24 (207.87–582.06)	1.44 ± 0.17	1.83	3.2	1.5
	2016-r16	545	22.43 (13.73–34.36)	410.67 (240.16–846.43)	1.02 ± 0.11	3.60	1.7	1.8

^(a)^ The pathogenicity of CpGV-M on susceptible larvae is used as a reference level; ^(b)^ Results from [2]

As a consequence, the first paragraph in the Discussion section must also be changed:

“Codling moth resistant natural populations did not respond to control by CpGV-M. The resistance levels were variable, from a hundred-fold to more than a thousand-fold resistance as a function of the relative frequency of the resistant genotypes. The RGV resistant colony, developed from a natural population, exhibits a homogeneous resistance level against CpGV-M higher than 10^5^ (for LC_50_) compared to the level for the susceptible colony.”

We apologize to the readers of *Viruses* for any inconvenience this may have caused.

## References

[1] Graillot B., Berling M., Blachere-López C., Siegwart M., Besse S., López-Ferber M. (2014). Progressive adaptation of a CpGV isolate to codling moth populations resistant to CpGV-M. Viruses.

[2] Berling M., Blachere-Lopez C., Soubabere O., Lery X., Bonhomme A., Sauphanor B., Lopez-Ferber M. (2009). *Cydia pomonella* granulovirus genotypes overcome virus resistance in the codling moth and improve virus efficiency by selection against resistant hosts. Appl. Environ. Microb..

[3] Asser-Kaiser S., Fritsch E., Undorf-Spahn K., Kienzle J., Eberle K.E., Gund N.A., Reineke A., Zebitz C.P.W., Heckel D.G., Huber J. (2007). Rapid emergence of baculovirus resistance in codling moth due to dominant, sex-linked inheritance. Science.

